# Quorum Sensing and Phytochemicals

**DOI:** 10.3390/ijms140612607

**Published:** 2013-06-17

**Authors:** Filomena Nazzaro, Florinda Fratianni, Raffaele Coppola

**Affiliations:** Institute of Food Science, ISA-CNR, Via Roma 64, Avellino 83100, Italy; E-Mails: fratianni@isa.cnr.it (F.F.); direttore@isa.cnr.it (R.C.)

**Keywords:** quorum sensing, phytochemicals

## Abstract

Most infectious diseases are caused by bacteria, which proliferate within quorum sensing (QS)-mediated biofilms. Efforts to block QS in bacteria and disrupt biofilms have enabled the identification of bioactive molecules that are also produced by plants. This mini review primarily focuses on natural QS inhibitors, which display potential for treating bacterial infections and also enhance the safety of food supply.

## 1. Introduction

During their evolution, bacterial communities have developed several sophisticated ways of interacting and associating with the environment they inhabit. Microorganisms may respond to a perceived change by altering their phenotype so that their metabolism and other activities are successful in the new environment. In the last few decades, it has also become evident that bacteria coordinate both bacterium-bacterium interactions and associations with higher organisms through intercellular communication systems that are often based on the expression of new genes, known as quorum sensing (QS) systems. QS-controlled behaviors take place only when bacteria reach a specific cell density. Such behaviors are unproductive if undertaken by a singular bacterium but become effective when the action is simultaneously performed by a group of bacteria. QS can regulate a number of activities, such as bioluminescence, virulence factor expression, sporulation, biofilm formation and mating. QS is realized through the bacterial production of chemical signaling molecules, also known as autoinducers or bacterial pheromones. These signals are produced while the bacterial population grows until a threshold concentration perceived by the bacteria is reached, resulting in the activation or repression of specific genes. The accumulation of a stimulatory amount of such molecules can occur only when a specific, sufficient number of cells, referred to as a quorum, are present [1].

The QS system can be seen as being based on the following crucial elements:

1. The autoinducers;
2. The signal synthase;
3. The signal receptor;
4. The signal response regulator;
5. The regulated genes (which form the so-called QS regulon).

The autoinducers can differ depending on the bacteria. They can be classified into three subclasses [2]: Autoinducers belonging to the first class are used by Gram-negative bacteria; the molecules of the second class are used by Gram-positive bacteria; and the third class of biomolecules can be used both by Gram-positive and Gram-negative bacteria.

**Figure 1:**
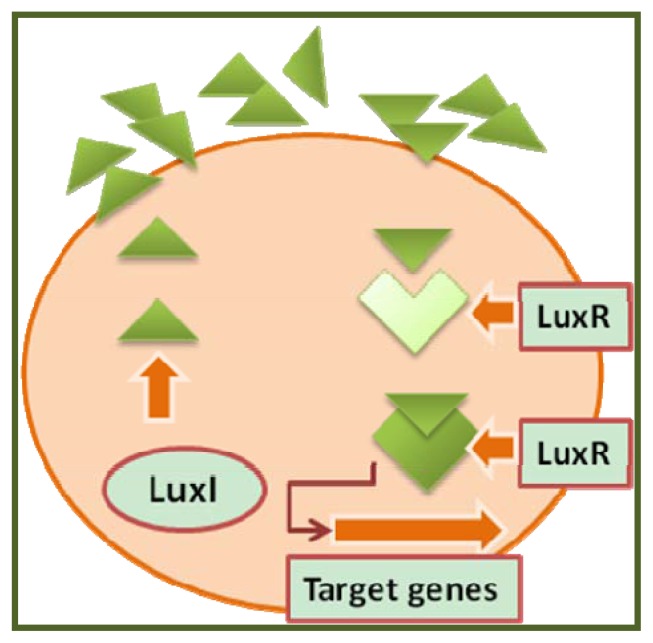
A typical quorum-sensing system in Gram-negative bacteria.

Gram-negative bacteria generally produce acylated homoserine lactone (AHL) as autoinducers, which are synthesized by a *LuxI*-type enzyme (signal synthase), encoded by the first gene of the lux operon, as shown in Figure 1. At a low bacterial cell density, the low level of transcription of the lux operon is insufficient for the activation of *LuxR*. When the cell density increases and signal levels reach a specified threshold level, *LuxR* activation can take place. The *LuxR*/3-oxo-C6-HSL complex thereby activates transcription via the *lux* operon promoter, giving rise to the expression of other genes, including (in *Vibrio fischeri*) *lux* AB genes encoding luciferase and *lux* CDE, which encodes the enzymes that produce the substrate for luciferase and, hence, bioluminescence. These molecules passively diffuse through the bacterial membrane and accumulate both intra- and extracellularly in proportion to the cell density. Quorum-sensing circuits have been identified in over 25 species of Gram-negative bacteria. Using the quorum-sensing mechanism, Gram-negative bacteria can efficiently couple gene expression to fluctuations in cell population density. Among these species, the *V. fischeri*, *Pseudomonas aeruginosa*, *Pseudomonas fluorescens*, *Pseudomonas chlororaphis*, *Erwinia carotovora* and *Agrobacterium tumefaciens* systems can be considered the best understood. More recently, the quorum system mechanism of *Chromobacterium violaceum* has been studied and used to evaluate the quorum and antiquorum activity of its biocomponents [3]. In the opportunistic human pathogen *P. aeruginosa*, a hierarchical *LuxI/LuxR* circuit regulates QS. Two pairs of *LuxI/LuxR* homologues, *LasI/LasR* and *RhlI/RhlR*, exist in *P. aeruginosa*. Both *LasI* and *RhlI* are autoinducer synthases, which catalyze the formation of the HSL autoinducers *N*-(3-oxododecanoyl)-homoserine lactone and *N*-(butryl)-homoserine lactone, respectively. These two regulatory circuits act in tandem to control the expression of a number of *P. aeruginosa* virulence factors. The *P. aeruginosa* quorum-sensing circuit functions as follows: At a high cell density, *LasR* binds its HSL autoinducer, and together, they combine with promoter elements immediately preceding the genes encoding a number of secreted virulence factors that are responsible for host tissue destruction during the initiation of the infection process. These pathogenicity determinants include elastase, encoded by *las*B, a protease encoded by *las*A; ExotoxinA, encoded by *tox*A, and alkaline phosphatase, which is encoded by the *apr*A gene. The *LasR*-autoinducer complex also activates the expression of the second quorum-sensing system of *P. aeruginosa* through the activation of a second class of specific target genes, encoding the stationary phase sigma factor rhamnosyl transferase, which is involved in the synthesis of the biosurfactant/hemolysin rhamnolipid, as well as genes involved in pyocyanin antibiotic synthesis and the *le*cA gene, which encodes a cytotoxic lectin [4]. In *Cromobacterium violaceum*, a Gram-negative microorganism found in water and soil, the phenotypic response to AHLs involves the production of a variety of factors, including antibiotics, hydrogen cyanide, proteases, chitinase and particularly violacein, a water-insoluble purple pigment with antibacterial activity [3,5].

## 2. Quorum System in Gram Positive Bacteria

**Figure 2:**
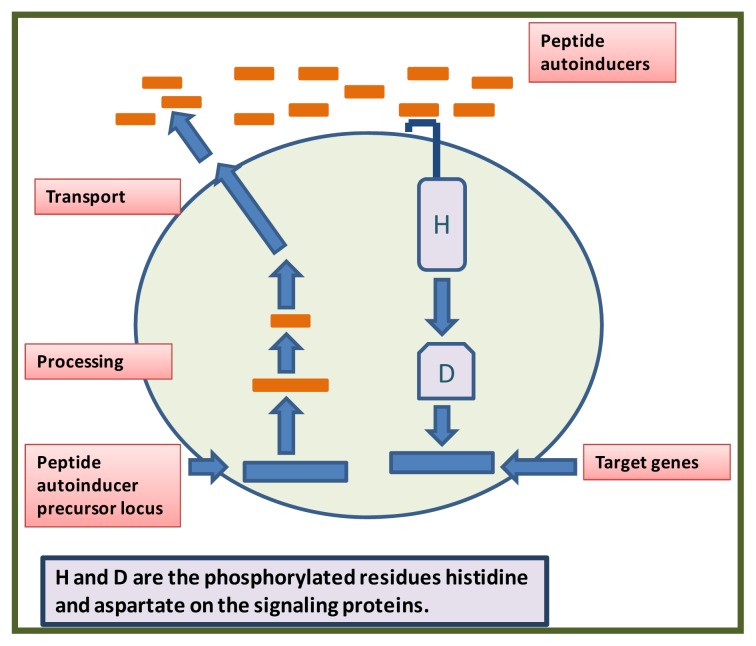
A typical quorum-sensing system in Gram-positive bacteria.

Gram-positive bacteria also regulate a variety of processes in response to an increasing cell population density, but in contrast to Gram-negative bacteria, which use HSL autoinducers, Gram-positive bacteria employ secreted peptides processed from precursors, that can be used as autoinducers for QS (Figure 2). Signals are actively exported outside the cell, where they interact with the external domains of membrane-bound sensor proteins. The transduction of a signal generated by a phosphorylation cascade culminates in the activation of a DNA-binding protein, that influences the transcription of specific genes, so that each sensor protein is highly selective for a given peptide signal. In Gram-positive bacteria, a peptide signal precursor locus is translated into a precursor protein that is cleaved to produce the processed peptide autoinducer signal. This signal is then usually transported out of the cell via an ABC transporter. When the extracellular concentration of the peptide signal accumulates to the minimal stimulatory level, a histidine sensor kinase protein belonging to a two-component signaling system detects it. The sensor kinase autophosphorylates a conserved histidine residue (H), and the phosphoryl group is subsequently transferred to a cognate response-regulator protein. The response regulator is phosphorylated on a conserved aspartate residue (D), which activates the transcription of a target gene or genes [4]. Similar to Gram-negative bacteria, Gram-positive bacteria can employ multiple autoinducers and sensors. However, some peptides can exclusively act from outside the bacterial cell, eliciting a specific set of gene expression changes by being transported into the cell, where they can activate a different set of behavioral changes. A furanosyl boronated diester molecule termed AI-2 and a non-boronated diester molecule, vA1-2, are used by Gram-positive and Gram-negative bacteria for intra- and interspecies communication [1]. Many Gram-negative bacteria use AHL autoinducers and also produce AI-2. Likewise, many Gram-positive bacteria display oligopeptide signaling systems as well as AI-2. Making and responding to combinations of these and potentially other types of chemical signals could allow bacteria to take a census of their own population nitrogen levels as well as the population density of other species in the vicinity. A distinct response to each signal, or a response based on a combinatorial sampling of a variety of signals, could enable bacteria to continuously modulate their behavior depending on the species present in a consortium.

## 3. Antiquorum Sensing Activity of Phytochemicals

The role of QS in food ecology has been investigated mainly in the last years [6,7]. In some studies, various signaling compounds, including AI-1 and AI-2, have been found to be present and even particularly concentrated in different foods, such as milk, meat and vegetables [8]. Little is known about the influence of food processing and storage on the production and release of these molecules by bacteria. As food matrices are generally solid environments (with the exception of liquid foods, of course), they give rise to microbial cells that become entrapped and localized at high densities at certain points, where they can show increased rates of growth and form biofilms. The presence and/or growth of a certain species, as well as its pathogenic activity and biofilm formation, will be affected by the presence of other microbial species and by all cell-cell interactions occurring in the solid food matrix, thus influencing the capability of each species to produce QS signals. The results from several reports suggest that targeting QS could be a new strategy for fighting biofilm infections [9]. Phytochemicals from fruits and vegetables are also capable of inhibiting QS-related processes in human pathogens [10,11], which is a particularly appealing property. For example, plant food-associated with QS activity may be of therapeutic interest, as the regular presence in the diet may positively affect the intestinal microbiota, preventing the concurrent invasion of pathogens. The continuing search for novel antimicrobial and antipathogenic agents has focused on exploiting the fact that plants surviving in an environment with a high bacterial density may possess protective mechanisms for combating infections [12]. Based on this argument, researchers are increasingly investigating herbal products in the quest for new therapeutic and antipathogenic agents that might act as nontoxic inhibitors of QS, thus controlling infections without encouraging the appearance of resistant bacterial strains [13]. In the current literature, it is estimated that while 10% of all terrestrial flowering plants have been used by different communities for treating diseases, only approximately 1% have gained recognition and been validated [14]. Thus, phytochemicals may represent the richest available reservoir of novel therapeutics [15]. Although the antimicrobial activities of plant extracts are beyond doubt, in many instances, their exact mechanism of antimicrobial functionality is not well understood [16,17].

Several types of QS-inhibiting phytochemicals, such as polyphenols, are capable of affecting biofilm formation in some bacteria [18–21]. However, the antiquorum sensing activity of herbal plants is still poorly understood, and it is very likely that it will be found that the antimicrobial efficacy is mediated by QS inhibition. The plant kingdom is a well-known source of medicines and contributes extensively to the development of pharmaceuticals [22]. Several quorum-quenching molecules have been identified from plants (Table 1). Many plants have co-evolved and established tightly regulated symbiotic or syntrophic associations with bacteria. Thus, it is not particularly surprising that higher organisms are capable of perceiving and responding to these molecules [10,23,24]. Both extracts and individual molecules from various types of fruits, herbs and spices have shown an ability to inhibit QS [11,19,25,26]. The best-investigated example of a eukaryotic organism that is capable of producing metabolites that specifically interfere with bacterial communication is the Australian red alga *Delisea pulchra*, which produces metabolites known as halogenated furanones. These compounds show a wide range of biological activities, including displaying antifouling and antimicrobial properties [27,28]. Certain furanones specifically interfere with AHL-regulated processes [29] by accelerating the degradation of the AHL receptor protein [30]. Teplitski *et al.* [23] demonstrated that several plants secrete substances that mimic bacterial AHLs and subsequently affect quorum-sensing-regulated behaviors in the bacteria associated with these plants. Thus, the detection of anti-pathogenic phytochemicals that inhibit the QS regulation of bacterial colonization and virulence factor production may provide very promising alternative anti-infective agents [31,32]. Plant extracts can act as QSIs due to the similarity of their chemical structure to those of QS signals (AHL) and/or their ability to degrade signal receptors (*LuxR*/*LasR*) [11,24]. GABA (γ-aminobutyric acid), which is produced by some plants, acts to promote the degradation of the OHC8HSL AHL signal by lactonase (AttM) in *A. tumefaciens*, thus limiting the QS-dependent infection process [33,34]. *Emblica officinalis*, through the presence of pyrogallol and its analogues, exhibits antagonism against AI-2 [35]. *Medicago truncatula* modulates *AhyR*, *CviR* and *LuxR* reporter activities in different organisms [22] as well as QS in general in *P. aeruginosa* and *S. meliloti* [36]. *Curcuma longa*, through the production of curcumin, inhibits the expression of virulence genes in *P. aeruginosa* PA01 [37]. Extracts from some varieties of apple (e.g., Annurca) and apple derivatives (e.g., cider) show demonstrated QSI activity, most likely due to the presence of different polyphenols, such as hydroxycinnamic acids, rutin and epicatechin, which act as AQS agents in synergistic manner against *C. violaceum* [38,39]. Antiquorum sensing activities have also been observed for extracts of *L. nobilis*, *S. oleraceus*, *R. officinalis*, *T. capensis*, *J. sambac*, *P. alba* and *P. nigra*, which are capable of decreasing violacein production [25]. Cinnamaldehyde and its derivatives affect a wide range of QS-regulated activities, such as biofilm formation in *P. aeruginosa* and AI-2-mediated QS in different *Vibrio* spp. [40,41]. Grapefruit, due to the presence of furo-coumarins, has been shown to inhibit the AI-1 and AI-2 activities of *V. harveyi* as well as biofilm formation by pathogens such as *E. coli*, *S. typhimurium* and *P. aeruginosa* [42]. Extracts of sour orange seeds containing limonoids, such as isolimonic acid, ichangin and deacetyl nomilinic acid 17 β-D-glucopyranoside, can cause >90% inhibition of AI-2 activity in *V. harveyi* at a concentration of 100 μg/mL and show activity against HAI- and AI-2-mediated bioluminescence [43]. Flavanones, flavonoids abundant in *Citrus*, have been shown to interfere with quorum sensing (QS) and affect related physiological processes [44]. Flavonoids, such as naringenin, kaempferol, quercetin and apigenin, inhibit HAI-1- or AI-2-mediated bioluminescence in *V. harveyi* BB886 and MM32. Flavanones (*i.e.*, naringenin eriodictyol and taxifolin) can be capable to significantly reduce the production of pyocyanin and elastase in *P. aeruginosa* without affecting bacterial growth. Naringenin and taxifolin also reduce the expression of several QS-controlled genes (*i.e.*, *las*I, *las*R, *rh*lI, *rh*lR, *las*A, *las*B, *phz*A1 and *rh*lA) in *P. aeruginosa* PAO1. Naringenin also dramatically reduce the production of the acylhomoserine lactones *N*-(3-oxododecanoyl)-l-homoserine lactone (3-oxo-C12-HSL) and *N*-butanoyl-l-homoserine lactone (C4-HSL), driven by the *las*I and *rh*lI gene products, respectively [45]. Quercetin, sinensetin, apigenin and naringenin display anti-biofilm formation activity against *V. harveyi* BB120 and *E. coli* O157:H7 [44,46]. Flavan-3-ol catechin can reduce the production of QS-mediated virulence factors, such as pyocyanin and elastase, and biofilm formation by *P. aeruginosa* PAO1 [47,48]. AHL-degrading abilities have been reported for a large number of legumes, including alfalfa, clover, lotus, peas and yam beans [49–51]. Biofilm formation by *E. coli* can even be disrupted by grapefruit juice and by rosmarinic acid produced by the roots of *Ocimum basilicum* (sweet basil) [11]. Phenolic plant secondary metabolites such as salicylic acid stimulate AHL-lactonase enzyme expression [52]. Ursolic acid at 10 μg/mL is capable of decreasing biofilm formation by 79% in *E. coli* and by 57%–95% in *V. harveyi* and *P. aeruginosa* PAO1 [53]. Aqueous extracts of edible plants and fruits such as *Ananas comosus*, *Musa paradiciaca*, *Manilkara zapota* and *Ocimum sanctum* have been found to show QSI activity against violacein production by *C. violaceum* and against pyocyanin pigment, staphylolytic protease and elastase production in *P. aeruginosa* PAO1 as well as its biofilm formation ability [54]. Broccoli extracts and its constituents can inhibit expression of QS-associated genes, thereby down-regulating the virulence attributes of *E. coli* O157:H7 both *in vitro* and *in vivo*, suggesting that this vegetal, like other Brassicaeae, has the potential to be developed as an anti-infective agent [55]. Other extracts, such as ethanol and ethyl acetate extracts from *Hypericum connatum*, exhibit QSI activity against *C. violaceum*, limiting its production of violacein [56]. Polyphenol compounds with a gallic acid moiety, such as epigallocatechin gallate, ellagic acid and tannic acid, which are commonly produced by many plants, are capable of specifically interfering with AHL-mediated signaling by blocking AHL-mediated communication between bacteria [19,20]. For example, pomegranates and berries are rich in ellagitanins such as punicalagin and ellagic acid, showing concentrations higher than 300 mg/100 gr [57]. In the gut, ellagitanins are hydrolyzed to ellagic acid by the microbiota and subsequently metabolized to form urolithin-A and urolithin-B; these metabolites can then accumulate in the human intestine, where they have important functions. Urolithin-A, for example is capable of modulating the growth of bacteria in rat intestines before and after chemically induced inflammation [58]. Urolithin A and urolithin B are also able to inhibit QS-associated processes (by up to 40%) and decrease the levels of AHLs produced by the entheropathogen *Y. entherocolitica* (by up to 45%) [59]. 4′,5′-*O*-dicaffeoylquinic acid (4′,5′-ODCQA) can act as a pump inhibitor with a potential of targeting efflux systems in a wide panel of Gram-positive human pathogenic bacteria [60]. Chlorogenic and vanillic acids and rutin, which can be found in berries, resveratrol (grapes), kinurenic acid (honey), daidzein (soy), dimethyl-esculetin (artemisia) and pomegranate extract can all be used as positive controls for QS inhibition [61]. Flavanols and proanthocyanidins form a complex with the spores and hyphae of pathogenic fungi of fruit crops [62], and phenolic polymer deposition is related to a decrease in bacterial multiplication rates. QS-inhibitory activity has been demonstrated for several unifloral honeys, which were found to be capable of inhibiting the production of AHLs: Chestnut honey showed the highest inhibitory activity, whereas orange and rosemary honeys were less effective. Truchado *et al.* [63] hypothesized that one of the factors affecting this inhibitory activity may be its floral origin, independent of the geographic location.

Different essential oils, also from ornamental plants, and their components have been observed to be effective against biofilms formed by *Salmonella*, *Listeria*, *Pseudomonas*, *Staphylococcus* and *Lactobacillus* spp. [64–68] Volatile organic compounds, such as those produced by rhizospheric strains *Pseudomonas fluorescens* B-4117 and *Serratia plymuthica* IC1270 may act as inhibitors of the cell-cell communication QS network mediated by AHL signal molecules produced by various bacteria, such as *Agrobacterium*, *Chromobacterium*, *Pectobacterium* and *Pseudomonas* [5]. Inhibition of bacterial QS may take place through different mechanisms including (1) inhibition of AHL synthesis; (2) inhibition of AHL transport and/or secretion; (3) sequestration of AHLs; (4) antagonistic action; and (5) inhibition of targets downstream of AHL receptor binding [11,22,36]. Biocontrol strategies for combating bacterial QS and biofilm formation through the use of natural phytochemicals can enhance the safety and security of foods and improve human health by reducing the capability of pathogens to invade the intestine, providing an opportunity for (1) downregulation of microbial spoilage activity and (2) altering microbial activity and survival. The first strategy for combating microbial spoilage activity presents the interesting advantage of preventing other undesirable microorganisms from colonizing a particular niche, while down-regulating the expression of enzymes such as proteases and lipases, resulting in a reduction in food damage. The second approach may be more desirable in the case of foodborne pathogens. The use of a bacterium’s own QS system against itself may minimize the possibility that the bacterium will adapt and become resistant to the applied QS inhibitors. Again, use of vegetal extracts and/or pure compounds can contribute to the limitation of food spoilage and the formation of bacterial biofilms. Extracts of garlic have been shown to block QS by *P. aeruginosa*, limiting the production of biofilms and thereby supporting the clearance of the bacteria [69]. Similarly, vanilla extracts are capable of interfering with QS in *C. violaceum*, suggesting that the consumption of vanilla-containing foods may be beneficial [70]. The presence of ascorbic acid, an AI-2 analog present in several fruits, may result in reduction of toxin production by *Clostridium perfringens* as well as decreased spore production when added to ground meat extract [71].

## 4. Conclusions

Interventions targeting bacterial QS in food are largely unexplored at present. Consumer expectations of obtaining pathogen-free food with an acceptable shelf life without the use of chemical additives represent a great challenge for the food industry based on currently available technologies. Biocontrol approaches using vegetal extracts or pure compounds obtained from plants offer some specific advantages compared to more conventional treatments. Indeed, thus-treated food is perceived as more “natural” and “green.” Although no one bio-control approach can currently address this problem in its entirety, such approaches, together, generate a toolbox of options that may act in synergistic and complementary ways, through various mechanisms of attack, that can be applied individually or in combination. Indeed, it is becoming increasingly evident that the use of phytochemical-based biocontrol strategies in food shows great potential for more realistic outcomes, although much work is still necessary to move these treatments from the laboratory bench to market.

Additionally, better understanding the potential of phytochemicals to inhibit QS activity is of great relevance to the field of research aimed at the identification and development of novel anti-QS compounds capable of preventing bacterial infections in humans. Such “antipathogenic” compounds, in contrast to antibacterial compounds, would not kill bacteria or completely stop their growth and would therefore most likely prevent the development of resistant strains [72]. Finally, in recent years, plant bioengineering could be helpful, which in particular, could influence their associated bacteria, as demonstrated for example by QS strategies suppressing the influence of pathogens of the genus *Pectobacterium* [73].

## Figures and Tables

**Table 1:** Phytochemicals with proved antiquorum sensing activity.

Molecules	References
Gamma aminobutyric acid (GABA)	Chevrot *et al.*, 2006 [33]; Zhang *et al.*, 2002 [34]
Pyrogallol	Ni *et al.*, 2008 [35]
Curcumin	Rudrappa and Bais, 2008 [37]
Cynnamaldheyde	Brackman *et al.*, 2008 [40]; Niu *et al.*, 2006 [41]
Furocoumarins	Girennavar *et al.*, 2008 [42]
Flavanones, flavonoids, flavonols	Truchado *et al.*, 2012 [44]; Vandeputte *et al.*, 2011 [45]; Vikram *et al.*, 2010 [46]; Vandeputte *et al.*, 2010 [47]; Rasamiravaka *et al.*, 2013 [48]; Leach *et al.*, 2007 [61]
Ursolic acid	Ren *et al.*, 2005 [53]
Rosmarinic acid	Vattem *et al.*, 2007 [11]
Salycilic acid	Yuan *et al.*, 2007 [52]
Epigallocatechin gallate, Ellagic acid, Tannic acid	Riedel *et al.*, 2006 [19]; Sarabhai *et al.*, 2013 [20]; Larrosa *et al.*, 2010a [57]
Urolithin A and B	Larrosa *et al.*, 2010b [58]; Gimenez-Bastia *et al.*, 2012 [59]
4,5-*O*-dicaffeoyl quinic acid	Fiamegos *et al.*, 2011 [60]
Chlorogenic acid, vanillic acid, proanthocyanidins	Leach *et al.*, 2007 [61]; Feucht *et al.*, 2000 [62]
Volatile organic compounds	Chernin *et al.*, 1998 [5]
Furanones	Manefield *et al.*, 2002 [29]
